# Instrumental and affective communication with patients with limited health literacy in the palliative phase of cancer or COPD

**DOI:** 10.1186/s12904-020-00658-2

**Published:** 2020-10-07

**Authors:** Janneke Noordman, Lotte Schulze, Ruud Roodbeen, Gudule Boland, Liesbeth M. van Vliet, Maria van den Muijsenbergh, Sandra van Dulmen

**Affiliations:** 1 Nivel (Netherlands institute for health services research), PO Box 1568, 3500 BN Utrecht, Netherlands; 2 Department of Tranzo Scientific Centre for Care and Well-being, Tilburg University, Tilburg, Netherlands; 3 Pharos, Dutch Centre of Expertise on Health Disparities, Utrecht, Netherlands; 4 Department of Health, Medical and Neuropsychology, Institute of Psychology, Leiden University, Leiden, Netherlands; 5 Radboud university medical center, Radboud Institute for Health Sciences, Department of Primary and Community Care, Nijmegen, Netherlands; 6 Faculty of Health and Social Sciences, University of South-Eastern Norway, Drammen, Norway

**Keywords:** Communication, Limited health literacy, Palliative care, Patients, Healthcare providers, Cancer, COPD

## Abstract

**Background:**

Patients have a ‘need to know’ (instrumental need) and a ‘need to feel known’ (affective need). During consultations with patients with limited health literacy (LHL) in the palliative phase of their disease, both the instrumental and the affective communication skills of healthcare providers are important. The study aims to explore instrumental and affective communication between care providers and LHL patients in the palliative phase of COPD or cancer.

**Methods:**

In 2018, consultations between LHL patients in the palliative phase of cancer or COPD and their healthcare providers were video-recorded in four hospitals in the Netherlands. As there was no observation algorithm available for this setting, several items were created to parameterize healthcare providers’ instrumental communication (seven items: understanding, patient priorities, medical status, treatment options, treatment consequences, prognosis, and information about emotional distress) and affective communication (six items: hope, support, reassurance, empathy, appreciation, and emotional coping). The degree of each item was recorded for each consultation, with relevant segments of the observation selected and transcribed to support the items.

**Results:**

Consultations between 17 care providers and 39 patients were video-recorded and analyzed. Care providers primarily used instrumental communication, most often by giving information about treatment options and assessing patients’ care priorities. Care providers assessed patients’ understanding of their disease less often. The patients’ prognosis was not mentioned in half the consultations. Within the affective domain, the care providers did provide support for their patients; providing hope, reassurance, empathy, and appreciation and discussing emotional coping were observed less often.

**Conclusions:**

Care providers used mostly instrumental communication, especially treatment information, in consultations with LHL patients in the palliative phase of cancer or COPD. Most care providers did not check if the patient understood the information, which is rather crucial, especially given patients’ limited level of health literacy. Healthcare providers did provide support for patients, but other expressions of affective communication by care providers were less common. To adapt the communication to LHL patients in palliative care, care providers could be less wordy and reduce the amount of information, use ‘teach-back’ techniques and pay more attention to affective communication.

## Background

Patients are known to have dual needs, i.e. the need to ‘know and understand’ (instrumental need) and the need to ‘feel known and understood’ (affective need), that demand instrumental and affective communication respectively [1, 2]. Instrumental communication is aimed at providing information and influencing patients’ understanding. Affective communication is directed towards patients’ emotions and asks for empathic behaviors. A patients’ need to know can be fulfilled by providing information about e.g. treatment options and prognosis, whereas a patients’ need to feel known can be satisfied by e.g. providing hope or reassurance [2]. For the outcome of the medical consultation, it is important that healthcare providers (HCPs) master both instrumental and affective communication skills and can adapt their communication to the dual needs of their patients.

Communication with patients with limited health literacy (LHL) in the palliative phase of their disease places additional demands on HCPs’ instrumental and affective communication skills. Almost 48% of the European population has limited health literacy [3], or 36% in the case of the Dutch population [4]. Health literacy is defined by Sørensen et al. [5] as *“the knowledge, motivation and competences to access, understand, appraise, and apply health information in order to make judgments and take decisions in everyday life concerning healthcare, disease prevention and health promotion to maintain or improve quality of life throughout the course of life”*. LHL patients have difficulty understanding healthcare information, applying this information to their situation, and asking questions about their disease. Although these patients ask fewer questions and take less control during consultations, they actually do wish to take part in decision-making as much as other patients [6]. However, as a result of insufficient communication between LHL patients and HCPs, these patients sometimes do not receive the care and medical information they need, which reduces their health outcomes [7].

Consultations in palliative care are often complex and loaded with unspoken emotional information. The palliative phase is defined as a condition in which there are no curative options left for the disease or when there is limited life expectancy [8]. About 4.4 million people worldwide die annually of serious conditions that are preceded by a palliative phase [9]. Although it is often said that people in this phase are terminally ill, patients can actually be in this phase for a long time (in the case of chronic obstructive pulmonary disease (COPD) or cancer it may be years). In general, patients depend heavily on the support and information about their disease from their HCP. It helps them to understand what is going on better and that might help them cope with their disease and to understand the consequences for their short or long-term future.

For most patients in the palliative phase of their disease, participation in decision-making [10] and good communication are the most important elements of end-of-life care [11]. Additionally, we would like to stress the importance of understandable person-centered information, especially for LHL patients [12]. Nevertheless, many HCPs do not check sufficiently whether patients understand the information provided, do not explore what information patients need, and rarely discuss their preferences for palliative or end-of-life care [13, 14]. A method for improving the way HCPs provide information and the patients’ understanding is the ‘teach-back’ method: the HCP asks the patient to relate what just has been discussed. If a patient understands the information, he or she should be able to teach the information back correctly to the care provider. A recent review also found that teach-back might be useful for HCPs during consultations with LHL patients in palliative care [15].

Besides instrumental communication, affective communication also plays an important role in preparing patients for their end of life. A trustful relationship between HCP and patient is important for patients to let them feel that they can discuss everything that is on their mind during the consultations [1]. Moreover, affective communication has been found to reduce anxiety and uncertainty during bad news consultations and enhances the recall of medical information [16–18]. Additionally, affective communication is more likely to decrease stress-induced physiological responses in the patient, as a result of the created positive affect, social support and trust from the HCP [19]. Using emotionally loaded words and utterances, such as empathic phrases, hope and reassurance, has a major positive impact on emotional distress, which is especially relevant within palliative care [16, 18, 20].

The present study explores the instrumental and affective communication of HCPs during consultations with LHL patients in the palliative phase of their disease. Cancer (especially lung cancer) and COPD are highly prevalent among LHL patients [21, 22]. The focus of this study is therefore on patients with cancer or COPD in the palliative phase of their disease. Insights into the communication process with this vulnerable patient group can provide input for designing communication-enhancing interventions for HCPs.

## Method

### Aim

This study aims to explore HCPs’ instrumental and affective communication with LHL patients in the palliative phase of COPD or cancer.

### Study design

An observational study was carried out in real-life clinical practice.

### Participants

Patients in four participating hospitals (three teaching hospitals and one general hospital, located in different regions in the Netherlands) were included in this study if they were aged 18 or older and understood Dutch. Only participants in the palliative phase of COPD or/and cancer were included. Additionally, they were only included if they had a lower level of education, for which the definition given by Statistics Netherlands was adopted [23] (i.e., no more than lower vocational level) and/or had LHL according to the three screening questions *“Filling in hospital forms is difficult. Do you agree?”*, *“Does someone help you fill out the doctor’s forms?”*, and *“How often do you need help for that?”* [24]. Patients were excluded if they answered ‘No’, ‘No’, and ‘Never’ respectively to these questions. HCPs (medical specialists and nurses) in the participating hospitals were included if they had a planned consultation with a LHL patient in the palliative phase of COPD or cancer.

### Data collection procedure

Consultations between LHL patients and HCPs were video-recorded between April and October 2018. Eligible patients were informed by phone a week before the planned visit to the hospital by a hospital coordinator or one of the researchers. If they expressed interest in participating, they were approached by a member of the research team in the waiting room just before seeing their HCP. If they did decide to participate, the inclusion criteria were checked and the patient signed an informed consent form before entering the consulting room, where an unmanned video camera was installed. HCPs were visible and audible on video, the patients were only audible. After the video-recorded consultation, patients received a gift voucher. Afterwards, the video recordings were stored in a secured locked room at Nivel that only the researchers had access to.

### Coding

As far as we know, there was no validated observation method for observational research within the palliative setting. Observational items were therefore operationalized by three authors (JN, LS and RR) to define HCPs’ instrumental and affective communication. Initially, there were seven instrumental communication items (part a) and three affective communication items (part b). These categories were based on the research protocol of a larger study called ‘A basic understanding’ (2017–2021) by five of the authors (SvD, JN, LvV, GB, and MvdM) and previous literature (e.g. [2]). During the observations, the main coder (LS) added three affective communication items after discussions with two of the authors (JN and RR); empathy, appreciation (of the person or situation) and emotional coping. The resulting final items, their definitions, and examples can be found in Table 1.

**Table 1:** Items for coding HCPs’ instrumental communication (a) and affective communication (b) during consultations with LHL patients in the palliative phase of cancer or COPD

**a. Instrumental communication**	**Definition**	**Examples (as defined a priori)**
1. Assessment of patients’ understanding of their disease	The HCP asks questions about the provided information, to see if the patient understood. Including the teach-back method.	“Do you understand what I just said?” “Could you maybe explain …”
2. Assessment of patient priorities	The HCP assesses and/or mentions the priorities and preferences of the patient.	“I’d like to know what’s important for you.” “I can imagine that you’d like to continue your swimming, wouldn’t you?”
3. Provision of information about current medical status	The HCP gives medical information and explains it to the patient.	“As we can see on the CT scan …” “Comparing these results with the last time …”
4. Discussion of information about treatment options	The HCP suggests one or more possible treatment options and explains them.	“Starting chemotherapy would be one possibility.” “I’ll explain the treatment options to you.”
5. Discussion of information about treatment consequences	The HCP discusses the consequences of a treatment, e.g. the side effects.	“It’s possible that you will feel nauseous after taking these medicines.”
6. Discussion of prognosis	The HCP discusses the expectations of the course of the disease within a certain time frame.	“The illness will probably become more active over time.” “These medicines will have an effect within three weeks.”
7. Giving information about emotional distress	The HCP gives information about the effect on emotions.	“It’s possible that you will feel lonely.” “Your situation may also influence your mental wellness.”
**b. Affective communication**	**Definition**	**Example**
1. Hope	The HCP sheds highlights the positive aspects of the situation.	“That looks very good!”
2. Support	The HCP emphasizes that the patient is not alone.	“We’ll do this together.”
3. Reassurance	The HCP tries to make the patient feel at ease.	“Don’t worry about that.”
4. Empathy	The HCP shows that he or she understands the patients’ situation.	“I can imagine that you are afraid, going through all this.”
5. Appreciation	The HCP shows that he or she appreciates the patient as a person/being.	“I really do respect the way you’re keeping going.”
6. Emotional coping	The HCP asks how the patient deals with their emotional distress.	“And what’s your response to this unpleasant situation?”

While observing the video recordings, relevant segments were noted along with the start and end times, and coded with the number of the corresponding item. Initially, only the first sentence of this segment was noted down to give an impression. Later, the most relevant segments were transcribed in full to support the findings.

### Reliability

The main coder (LS) observed all video-recorded consultations (*n* = 40) twice, with more than a day in between. A random selection of 10 consultations (25% of the total sample) were observed by a second coder (JN) to examine the reliability of the observations. All double-coded observations were discussed between these two coders to confirm that the items were based on the same concepts. One consultation was eliminated from the dataset as a result of inconsistencies between the two coders. This specific consultation was found to be relatively long and differed greatly from the others included in terms of the content (i.e., there was nothing about any medical conditions and it was more psychologically focused). In the resulting nine doubly coded consultations, 152 observations were found in total (by at least one of the two coders or both). The observations of the second coder matched those of the main coder in 86% of cases, whereas the observations of the main coder matched those of the second coder in 95% of cases; both percentages indicate good agreement between observers.

### Analysis

The frequencies for each consultation of all the coded items were analyzed using STATA-14. Background characteristics of patients and HCPs were added for each consultation: the function and gender of the HCPs; the gender, age, and educational level of patients; the answers to the three LHL questions; and whether or not there was another person present during the consultation to assist the patient. Finally, the duration of the consultation, the type of consultation (new, control or composite) and the patients’ disease were recorded. Descriptive analyses were performed on the above-mentioned variables in STATA-14. In order to substantiate the frequencies, the main coder looked through the various transcribed observations and picked out the most significant (initial sentences of the) quotes. This resulted in around three quotes per item that were then transcribed verbatim, after removal of any personal identifiers. After discussion with the second coder, some quotes were eliminated because they were vague without the context of the whole consultation or because of overlap with other quotes. The remaining quotes were used to substantiate the coded items.

## Results

### Participants

Seventeen HCPs participated in the study, of whom ten were women and seven were men. The HCPs were physicians (*n* = 10), physicians in training (*n* = 1) or nurses (*n* = 6). An average of two consultations were recorded per HCP (SD 1.21, range 1–5). In most of the consultations, there was one HCP present, except for two consultations with two HCPs involved. During six consultations, a physician in training attended the consultation but was not involved in the conversations.

A total of 39 patients were included, 21 men and 19 women (52.5 and 47.5%, respectively). In 33 consultations, the patient was accompanied by a companion (20 by their partner, 9 by their son or daughter and 4 by someone else). Of the included patients, 31 had a low level of education, 6 had a medium level and 2 a high level; in one patient, the educational level was unknown. Seven patients who said they were not LHL were nevertheless included based on the opinion of their HCP who considered them to have low health literacy or to be vulnerable in communication. Five of these had a lower level of education and two had a medium level. Thirteen patients were diagnosed with COPD, 26 were diagnosed with cancer and one patient with an anomaly of the lung.

### Consultations

The consultations had an average duration of 22.53 min (SD = 13.13; range 5.57–69.58). Three consultations were first (new) consultations, 29 were checkup visits and 8 were composites (i.e., a follow-up consultation in which the patient presented new problems or symptoms).

### Instrumental communication by HCPs

In general, HCPs primarily communicated instrumentally, spreading this across various items. Interestingly, the duration of the instrumental communication fragments was longer than the duration of the affective communication fragments during the consultations. The total number of each item is shown in more detail in Table 2. Additionally, a quote is given as an example of each item. More quotes related to the items can be found in Additional file, Annex A.

**Table 2:** Instrumental communication by HCPs: items discussed and number of times discussed

Instrumental communication items	Not discussed/provided (*n*)	Discussed/provided(*n*)	Mentioned once per consultation	Mentioned two times per consultation	Mentioned more times per consultation	Total number of times subject is discussed
1. Understanding	25	14	10	2	2	21
2. Priorities	7	32	8	10	14	87
3. Medical status	1	38	8	7	23	110
4. Treatment options	13	26	7	6	13	84
5. Treatment consequences	12	27	16	9	2	43
6. Prognosis	18	21	7	8	6	48
7. Giving information about emotions	28	11	10	1	0	12

#### Assessment of the patients’ understanding of their disease

In the majority of consultations (*n* = 25), patients’ understanding of their diseases was not assessed by the HCP. In most of the other consultations, this understanding was assessed once (*n* = 10). In total, this item was mentioned least often of all the observed instrumental communication aspects (21 times).

Checking if the patient understood the information let the HCP verify whether they had explained the information clearly to the patient and, if needed, they could then re-explain and check again. This could be attempted by questions such as ‘what did we discuss previously?’ or more elaborate by using the teach-back method mentioned earlier. In the quote below, the HCP asked the patient to summarize what they remembered about the last conversation. Additionally, the HCP explained the reason for asking.

*“Maybe it would be most convenient for me to go back to Friday’s conversation, if you would please summarize for me what I explained then, as we discussed a lot of material already then. Lots of information at once. So maybe it is nice for me to ( …*) *know what has stuck ( …*). *Then I know where to start, because oxygen is all linked to that. What can you remember about that?” (Z1L05).*

#### Assessment of the patients’ priorities

In most consultations (*n* = 10), the HCP mentioned the patients’ priorities twice. In eight consultations it was mentioned once, while in seven it was not mentioned at all.

HCPs assessed patients’ priorities for their treatment options and took into account the wishes and circumstances of the patient. The following quote is an example of a HCP who tried to involve the patient in decision-making. In doing so, the HCP provides space and time for the patient to think about their own priorities.

*“In the end, you decide with us, don’t you? Because it’s your body, so really think about it, what you want and what you don’t.” (Z1L03).*

Some HCPs also took the patients’ personal life into account. For instance, they asked about patients’ daily affairs (like work or hobbies) to make sure that certain decisions fitted in their daily life.

*“Because you have a job, don’t you? How many days do you work?” (Z3L03).*

#### Provision of information about current medical status

In most consultations (*n* = 11), information about the patients’ current medical status was provided three times by the HCP. This item of instrumental communication was therefore mentioned the most often in all consultations (110 times). In one consultation, this information was not provided. The reason seemed to be the limited time of the consultation. Moreover, the purpose of that particular consultation was to check if certain complaints were present, which was not the case.

Almost all HCPs provided information about the medical status and development of the patients’ disease. Most HCPs presented this information to the patient using their computer screen at the beginning of the consultation. During this item the HCPs spoke most of the time; the patients sometimes asked questions in between or afterwards. In the following example, the HCP explained the results of a CT scan to the patient.

*HCP: “What you see in the blood is that the CEA, the substance that makes the cancer, is rising. Well, it’s 8.9 now and last time was 6.6 and, it’s come up from 2.4 around this time last year. And we did that CT scan, which shows that the cancer is still growing.”*

*Patient: “OK. Everything?”*

*HCP: “All the spots in the lungs have gotten a little bigger. There is a larger abnormality in the lungs too. It was about two centimeters; it has now become three centimeters. In particular the largest tumor, which is the most obvious, has also grown. And all those other small spots that were only a millimeter in size, well, they’ve also grown a few millimeters. But especially the tumor that went from two to three.” (Z3O04).*

Sometimes the HCP repeated or summarized what had been discussed in previous consultations, especially when the patient could not remember (or only partially) or could not teach back the information provided (see item 1). See for example the quote below from a HCP summarizing the disease history of the patient and what was discussed during a previous consultation.

*“To summarize: she was admitted with pneumonia. We have treated it, but we also know that she has COPD, of course. So you’ve been smoking through the years and your lungs have worn out, as it were. Lung damage has occurred that can no longer be made better. And that means that at the moment there is an infection in which the respiratory tract is continuously too irritated – it’s a chronic condition. And if anything comes on top of that, the whole house of cards collapses. That’s accompanied by more mucus, stuffiness, things like that. What I explained last time is that there is a difference between the way the lungs function in a healthy person and in someone with COPD.” (Z1L05).*

#### Discussion of information about treatment options

Information about treatment options was not discussed in 13 consultations. We do not however know which treatment options were still available for patients. When treatment options were discussed, they were mentioned once in most consultations (*n* = 7).

In a number of consultations, HCPs discussed the options for starting a treatment to minimize certain complaints. This could be a medically invasive option such as surgery or chemotherapy, but it could also be additional to the current treatment, such as psychological help or physiotherapy. In the example below, the HCP listed different treatment options that the patient could take into consideration for dealing with their somatic and psychological complaints.

*“What you can consider, of course, is pulmonary rehabilitation. That should be possible, and it’s also an option in itself. You can do that at home in primary healthcare, as they say. For example, twice a week with a physiotherapy trainer. You can do it here in the hospital, three times a week; that’s a somewhat heavier program. And then not only physiotherapy, but also dietetics, possibly the psychologist, social work if necessary, the lung nurse, who is continuously present. Occupational therapy if necessary, to see if you still need medical aids. You can do that. Or you go to what we call tertiary rehabilitation, but then you should think of the rehabilitation center. I think you could get started with that too. But you have to be able and willing to do that. But we can talk about that next time; maybe we should talk about that depending on those results.” (Z3L02).*

#### Discussion of information about treatment consequences

In most of the consultations (*n* = 27), the information about treatment consequences was given once (*n* = 16), or twice (*n* = 9). In 12 consultations, no treatment consequences were given by the HCP. However, it is not known in how many consultations talking about a treatment option was possible. From another observation within the same study (Roodbeen et al., submitted) it was concluded that in 36 of all 40 consultations a treatment decision was made.

When information about available treatments was provided to a patient, the HCP could outline the consequences of this specific treatment by addressing the advantages and disadvantages. In the quote below, the HCP discussed one advantage and one disadvantage of inserting a stent, which helps the patients to make an informed decision about the treatment.

*“The advantage of a stent is that food can pass very quickly, because it just makes room right away. The downside of a stent is that it doesn’t do anything against malignant cells, does it? It only makes space. And if you wait over time, the active disease, the malignant disease, can start to grow again. So that also reinforces … ( …) Well, those are disadvantages, aren’t they?” (Z2R06).*

The treatment consequences also included potential reasons for not choosing a certain treatment, as in the following quote. This quote is also an example of one of the few times that HCPs talked with the companion instead of the patient.

*“I’m a bit afraid of that if I give that for a long time, that she could get complaints.” (Z1L05).*

#### Discussion of prognosis

The patients’ prognosis was not discussed in nearly half of the consultations (*n* = 18). When the prognosis was discussed, it was often discussed twice (*n* = 8). However, we do not know whether the HCPs had enough information to make a correct prognosis for all patients in every consultation. Even when enough information is available, it is hard for HCPs to make a correct prognosis. Additionally, we do not know whether patients were already aware of their prognosis or if they wanted to discuss it during the recorded consultation.

HCPs provided prognostic information about how the patients’ disease was expected to develop. For instance, the chance of successful treatment or how a medicine works was discussed. Sometimes the HCPs mentioned other patients’ experiences with the same treatment. In the example below, the HCP drew a picture of how the patients’ disease might develop.

*“However, the disease is slowly getting more active. And one of the things that you see happening most often is that people often get tired. People get … when you have cancer, you hear very often that people get tired. Your battery … your battery does not recharge properly, it can no longer charge properly. Put it this way: everyone has a battery – that’s how I see it as a doctor, right? And well, and then people rest more. Suppose you suddenly get anemic – you can get that theoretically. Well, if that’s bad and you’re still fairly mobile, then you get given some blood. To give an example.” (Z3L05).*

#### Giving information about emotional distress

Information about possible emotional distress was given in less than half of the consultations (*n* = 11). When discussed, it came up once per consultation, with one exception when it was discussed twice.

Besides the medical and treatment information, HCPs also provided information about the emotions (mostly negative) that could arise as a result of the patients’ disease. Contrary to affective communication about emotional coping, this item demonstrates the information that was given by the HCP about the emotions that the patient could possibly experience.

*“Because they also pay attention to the psychological side of things, for example. There are, of course, quite a lot of people who, well, if you have a chronic illness, you sometimes get down. Or get anxious. That isn’t crazy at all, of course.” (Z1L08).*

Additionally, some HCPs acknowledged the emotions shared by the patient and mentioned that their emotional reaction was normal.

*“Those are all things that will be very difficult now.”(Z1L07).*

### Affective communication

The affective communication by HCPs differed, as can be seen in Table 3. Most HCPs supported for their patients, but other affective communication by HCPs was less common. More quotes on affective communication can be found in Additional file, Annex B.

**Table 3:** Affective communication by HCPs: items discussed and number of times discussed

Items of affective communication	Not discussed/ provided (*n*)	Discussed/ provided(*n*)	Mentioned once per consultation	Mentioned two times per consultation	Mentioned more times per consultation	Total number of times subject is discussed
Hope	22	17	12	4	1	23
Support	8	31	12	7	12	77
Reassurance	23	16	7	5	4	29
Empathy	21	18	9	5	4	38
Appreciation	30	9	8	1	0	10
Emotional coping	25	14	9	3	2	21

#### Hope

In most of the consultations (*n* = 22), hope was not mentioned by HCPs. When hope was mentioned, it was most often mentioned once (*n* = 12).

HCPs try to give some positive possibilities for the near or longer-term future to the patient. They say for instance that they hope the medicine will work, or that the treatment will not produce too many side effects. The following quote illustrates hope introduced by the HCP.

*“Well, we’re going to try to stop it. We’re hoping to make it smaller and there’s a very good chance of that. And then we hope it stays quiet for a long time.” (Z3O04).*

Hope was also provided without mentioning the word itself, like in the following quote.

*“We assume that we’re done. We’re assuming that you are now clean.” (Z2L06).*

#### Support

Overall, support was mentioned most often of all the affective communication items (78 times in total). HCPs mentioned support once in most of the consultations (*n* = 12), with a maximum of seven times in one consultation. In eight consultations, no support was offered.

Offering support lets the HCP emphasize that the patient is not left on his or her own, or that the HCP (and the whole medical team) will support the patient as much as possible. The word ‘we’ is important in this case because it focuses on the collaboration between HCP and patient. For this item to be coded, the word ‘we’ had to suggest support from the care provider and not just a generalized use (e.g., “Did we have a good week?”). This support could help build a relationship in which the patient feels more at ease and supported in their illness, as is illustrated in the following quote.

*“We are all here to keep you alive for as long as possible on the one hand, and to keep you alive as best as possible on the other.” (Z2L08).*

#### Reassurance

Most of the consultations (*n* = 23) did not contain any reassurance by the HCP, but 16 did, mostly once in each consultation. HCPs tried to overcome unrealistic (negative) thoughts or feelings of patients by reassuring them, e.g. by correcting negative assumptions about the treatment or medication. Whereas *hope* concentrates on positive feelings for the future, *reassurance* focuses on reforming the negative feelings into positive feelings in the present, as shown in the following quote.

*“We don’t want to irradiate you so often, you know.” (Z2R07).*

In the quote below the HCP tried to ease the patients’ concerns and fears.

*“So that’s it again: every cloud has a silver lining, so to speak.” (Z2R06).*

#### Empathy

HCPs did not show empathy verbally to patients in more than half of the consultations (*n* = 21). When present, it was most often given once (*n* = 9) or twice (*n* = 5).

Empathy lets the HCPs show that they acknowledge the patients’ psychological or emotional feelings or practical experiences, as is illustrated in the following quote.

*“Yes, I can imagine: not conducive to sleep at all.” (Z4A04).*

#### Appreciation

In these consultations, most HCPs did not verbally express appreciation of the patient; it was not mentioned in 30 consultations. When mentioned, it was done mostly once (*n* = 8) per consultation. Overall, this item was mentioned least (10 times) out of all the affective communication items.

Examples of appreciative comments were confirmation that the patient was acting bravely or respect for the patients’ hard work. An example of an appreciative remark for the patient is shown below.

*“I always admire you, because you always try to do as little as possible.” (Z4A04).*

Appreciation could also be shown about the medical situation rather than the patient, as in the following example:

*“I am actually also satisfied. I am glad that it has recovered so much.” (Z1L10 & 11).*

#### Emotional coping

Emotional coping was not discussed in more than half of the consultations (*n* = 25). When discussed, this was mostly done once (*n* = 9).

The HCP could ask about the way the patient deals with all the emotional distress, as it is shown in the following quote.

*“Well, that’s very difficult because … well, what do you do at such a moment?” (Z3L02).*

Some HCPs explicitly asked how the patient or family was feeling. They wondered how the patient was dealing with emotional or other difficulties, as for example in the quote below where a HCP spoke to a patient whose wife has passed away recently.

*“How are you getting on now that you’re on your own?” (Z4A06).*

It is worth noting the fact that some HCPs also addressed the emotional coping by the patients’ companion. This happened once in two consultations.

## Discussion

This study aimed to explore the instrumental and affective communication between HCPs and LHL patients in the palliative phase of COPD or cancer. Our study showed that HCPs provided mostly instrumental communication to their LHL patients. Information about treatment options was mentioned most often, in almost all consultations. Moreover, our results show that most HCPs did not check patients’ understanding and did not use the teach-back method.

Zooming in on the specific quotes about instrumental communication (especially concerning information provision about the patients’ medical status), we believe the HCPs often provided too much information, using difficult words and lengthy sentences that were not adapted to LHL patients. This ‘wordiness’ (using more and more difficult words than actually needed) could have resulted in information being provided unclearly to LHL patients. Although we did not ask about the needs and understanding of the provided information of patients in this specific study, we do know that LHL patients can on average remember a maximum of three subjects per consultation [25]. Additionally, previous research has shown that cancer patients as a whole (not only LHL patients) forget more than half the information provided (about diagnosis, prognosis, and treatment), independently of their age [26].

In contrast, we were pleased to find that HCPs assessed patients’ priorities in the majority of the consultations. This indicates that HCPs take account of the wishes and circumstances of the patient. These findings differ from previous research, which found that HCPs do not always check the patients’ priorities or what is important for their patients [13, 14]. As mentioned before, the majority of patients in the palliative phase do want to participate in treatment decisions [10] and list good communication as one of the most important elements of end-of-life care [11]. Recent research has shown that LHL patients also want to participate in decision-making, but need the support to do so [12].

Another finding is that patients’ prognoses were not mentioned during almost half of the consultations. Previous research has shown that many cancer patients have unresolved questions about their prognosis and do not understand the prognosis provided [27]. However, not all patients want to discuss their prognosis in every consultation [28]. Additionally, many oncologists avoid discussing the prognosis or provide rather optimistic results [29]. Giving a prognosis for COPD patients is more difficult than for cancer patients as a result of the lengthy course of COPD. This also hinders communication about the end of life for these patients [30] and probably especially for LHL patients as they need clear information (e.g. short sentences, no jargon) in the present tense [31]. Another possible explanation for our findings could be that the prognosis has already been discussed with the patient in an earlier consultation, or will be discussed in a subsequent visit. As mentioned before, it is important that HCPs ask patients (not only LHL patients) about their wishes regarding prognosis information, and adapt their instrumental and affective communication accordingly.

With respect to affective communication, most of the HCPs did provide supportive words to their patients. Other affective communication, such as hope, reassurance, empathy, appreciation, or discussing emotional coping, were observed less often. Previous research among COPD patients has shown that patients want to receive emotional support and discuss end-of-life care with their physicians [30]. According to a scoping review [32], the emotional engagement of a HCP could empower, enable, and encourage the patient to take control of their disease. Focusing specifically on LHL patients, a survey study by Chu and Tseng [33] suggested that HCPs’ empathy could help LHL patients understand preoperative information better. The authors therefore claim that improving empathic communication should get priority, especially for LHL patients. Although it is sometimes difficult to find the balance between providing hope and being realistic, reassurance provided by oncologists and pulmonologists could decrease patients’ uncertainty and anxiety and could also increase their self-efficacy and satisfaction [34]. According to Olsman et al. [35], there are three perspectives of hope that could improve HCPs’ communication: the realistic, the functional and the narrative perspectives. Hope could not only be given about the outcomes of the illness itself, but also about side effects or someone’s wellbeing. Additionally, appreciation of the patient and their behavior and understanding their identity and fears are found to be very important factors in supporting patients [36].

Furthermore, we found that instrumental communication fragments were longer than affective communication fragments. This suggests that instrumental communication takes up the larger part of the consultation while affective communication aspects receive only seconds or perhaps minutes. This does not necessarily have to be a problem. For example, the study by Fogarty and colleagues [37] showed that 40 s of compassion could have a positive effect on the anxiety of female patients with breast cancer. This finding suggests that affective communication does not have to take up a lot of time within the consultation. However, these patients were not screened for health literacy. So the question arises of whether this is different in consultations with LHL patients. More research is needed to understand the optimal duration of affective communication and these time differences and what they mean.

### Strengths and limitations

To our knowledge, this is the first study to explore the instrumental and affective communication of HCPs with LHL patients in the palliative phase of cancer or COPD by analyzing real-life consultations. We have created several items that we believe collect relevant data about the communication skills of HCPs in this setting and these were coded reliably.

Some limitations are worth mentioning. Firstly, although the coded items provided relevant data about the communication and these items were coded reliably, we did not validate the items. In addition, the division between instrumental and affective communication is somewhat artificial and not always evident, i.e. these are theoretical concepts that can overlap in practice.

Secondly, we focused on positive expressions by HCPs. We did not code a lack of expressions, e.g. not showing empathy or not discussing treatment options (so called ‘missed opportunities’). Moreover, as we only coded verbal affective communication, the HCPs may possibly have used affective expressions non-verbally. Their reactions also depend on the non-verbal expressions of their patients, who were not visible.

Thirdly, the HCPs were aware that our study was focusing on their communication with LHL patients. This could have influenced their behavior. They were, however, unaware of the specific focus on instrumental and affective communication.

Fourthly, there was sometimes disagreement between the patients’ and HCPs’ assessments of the patients’ level of health literacy. This might be related to the fact that patients with limited health literacy often hide the fact that they do not understand health information because they are ashamed [12, 38]. In addition, due to time constraints or patients’ emotional overload, eight patients were not asked the three screening questions. These patients were included based on the opinion of the HCP, which could have biased our population.

Fifthly, the coded video-recorded consultations were snapshots of daily practice. It is possible that topics that were not discussed in the recorded consultation had already been discussed in a previous consultation or would be discussed in a future one.

Because of the small sample size, it was not possible to look at differences between male and female patients, between the individual HCPs, between first and follow-up consultations, or between patients with cancer or COPD. However, these differences could have influenced the instrumental and affective communication of the HCPs.

Lastly, the items identified could be based on the coders’ personal and cultural values and backgrounds, especially for affective communication. For instance, previous research has shown that oncologists think the definitions of reassurance and hope overlap to some extent [39].

### Recommendations for research and practice

To adapt the communication to LHL patients in palliative care, HCPs could reduce their ‘wordiness’ and simplify their language (i.e., use short sentences in the present tense) and the amount of information (i.e., discuss a maximum of three subjects [25]), use the teach-back technique and pay attention to affective communication, especially as previous research found that empathy could help patients to understand the information better [33].

Future research could focus on the appropriate amount and quality of information provision and affective communication according to both LHL patients in palliative care and HCPs. The understanding and recall of information by LHL patients of this current study will be addressed as part of the larger study (‘A basic understanding’).

## Conclusion

HCPs provided mostly instrumental communication, especially treatment information, in consultations with LHL patients in the palliative phase of cancer or COPD. Most HCPs did not check if the patient understood the information, which is rather crucial, especially given patients’ limited level of health literacy. HCPs did support the patients but other forms of affective communication by HCPs were less common. To adapt the communication to LHL patients in palliative care, HCPs could reduce their wordiness and simplify their language, reduce the amount of information, use the teach-back technique and pay attention to affective communication.

## Supplementary information

**Additional file 1.**

## Data Availability

The datasets used and/or analyzed during the current study are available from the corresponding author on reasonable request.

## Abbreviations

HCP: Healthcare provider
LHL: Limited health literacy
