# Supplementary material for: Instrumental and affective communication with patients with limited health literacy in the palliative phase of cancer or COPD
Source: BMC Palliat Care. 2020 Oct 7;19:152. doi: 10.1186/s12904-020-00658-2 (PMC7542099; doi:10.1186/s12904-020-00658-2)
Supplement: Supplementary file 1 — Additional file 1. [file 12904_2020_658_MOESM1_ESM.docx]

**Additional files**

**Annex A. Instrumental communication quotes**

HCP= Healthcare provider

P= Patient

**1. Assessment of the patients’ understanding of their disease**

“Do you get it? Yes, a lot of information!” (Z2L01)

“With those medicines, do you still have an overview?” (Z2R03)

**2. Assessment of the patients’ priorities**

HCP: “No, but it’s nice to know this because then we know your background. So, normally I’d have asked you what makes you happy in life. You say, ‘My work, and working with the elderly was my passion.’ That's how I hear you say that.”

P: “Yes. Yes.”

HCP: “In fact, you wouldn't want to stop working after the age of 65. But that would normally be my question. Normally, my question would be, ‘What do you enjoy in life?’ Well, that's about traveling. So you, you’re already mentioning things that I would like to know.” (Z4A03)

“That’s annoying of course, because then you’re already waking up five times in the night anyway. It’s sometimes possible that you may have to urinate regularly, and that’s an annoying complaint, I can imagine, because then you have to fall asleep again and again. Well, that’s difficult because you have pain on that side, so all that isn’t conducive to a good night's sleep, of course.” (Z4A04)

“Looking back, what are the most important things for you? Things that make you say that it’s not going well enough yet?” (Z4A02)

“That doesn't really matter to us. Most importantly, we need to know if you still think the conversations and visits are worthwhile. And as long as you say, ‘Yes, I like coming by once every few weeks or months,’ then that’s fine. And the moment you say, ‘Well, I don't feel like it for the time being; I’ll contact you again if necessary’ then that’s fine too.” (Z4A02)

**3. Providing information about the current medical status**

“Well, actually we don’t really know what’s going on, do we? On that scan that was made, which has been compared against the scan from February of course, and then against the scan from before January. The irradiation – the short irradiation that we did in January. And the radiologist then says to us, ‘I see that that piece, which had then thickened and was completely closed, has indeed become a bit smaller. But actually he’s still seeing a lot of mass there, there is still a lot of tissue. And the radiologist can’t tell on a scan if that’s scar tissue or if it’s still malignant tissue. And because there seems to be so much, the doctor and I think there really must be some malignant tissue in there: it really can’t all be just scar tissue.” (Z2R06)

“I’ll come straight to the point. That abnormality has grown a bit. (silence) So something is happening there and of course that means that we are not completely at ease with it. We’ve seen each other before, of course, and you went to see the doctor when I wasn’t there. And you should have been to the radiotherapist once, to another doctor. Because what we are dealing with is that spot you have on your lungs, which we think could very well be malignant but which we’re finding very difficult to get at in terms of both diagnostics and treatment, because your health has of course already deteriorated a lot in other areas. We’re left with lungs that are naturally no longer as they should be.”(Z1L03)

**4. Discussing information about treatment options**

“So I already discussed this with the pulmonologist this morning and he says yes, maybe we should keep that in mind, starting that oxygen already. And then, of course, the next question is how we proceed from there.” (Z1L05)

“I asked the nurses – we have specialist nurses here – what you had said on Friday, which will help us to make the best possible arrangements at home for a patient who has been admitted previously. This means that the nurse would discuss it with you or contact the type of housing where she is now – the sheltered housing, in other words. In particular, take a look at what the care is like there and what can be done there. And how can we… is that care enough for her? Because if the care there is enough, if we can scale it up, then you can probably go home soon. If that doesn’t work, we should check out how we are going to do it. Then I’ll have to consult with the pulmonologist about whether we choose to try rehabilitation, see if we can get you even better so that we can get you home with care, or whether we say that we don’t really have any hope, in which case we should think more about a nursing home or something like that.” (Z1L05)

“And then there are other painkillers that we can start for this, such as amitriptyline for example, which is a medicine for that, a medicine that you give for the night and that also helps people sleep better sometimes. So trying that would still be an option for you, and see if that helps make your complaints a little better. (Z4A04)

**5. Discussing information about treatment consequences**

“This [chemotherapy] also has a number of side effects. This chemotherapy doesn’t make you go bald, but it can of course make you nauseous again. You may vomit. We do give very good medication for that, so most people do get a bit tired and feel sick, but getting really nauseous, well, we don’t see that very often anymore. And if it does happen, I have even stronger medication to get it under control. So that almost always works. The other thing chemotherapy can do is suppress your bone marrow. The bone marrow is what’s inside your bones. It makes your blood, so your red blood cells, white blood cells, and platelets are all made there. And just when there aren’t enough red blood cells, we say that you are anemic, and you may need a blood transfusion. We hardly ever see that with these treatments.” (Z3O04)

HCP: “But you shouldn’t overestimate the effect of those valves.”

P: “What do you mean?”

HCP: “It’s not a miracle cure.”

P: “No, definitely not. And it doesn’t always work. It doesn’t always work; I’m aware of that.”

HCP: “OK.”

P: “Because the body can reject it.”

HCP: “Yes, anything can happen to it.”

P: “Anything can happen to it. You can get a collapsed lung.”

HCP: “Yes. Bleeding. An infection.”

P: “I know all of that.”

HCP: “Good. But we’re still going to try it?”

P: “And I still want to go for it.”

HCP: “OK.”

(Z1L12)

**6. Discussion of the prognosis**

“But at some point there will come a time when things are going less well, and the time will also come when you die; that’s the same for all of us. And it is good to get ahead of the game a bit, good to know where you could go then and what the possibilities are. Look, there are various ways in the Netherlands in which you can die. It can be suddenly, if you have an accident: you may not even have a chance to suffer if it happens very quickly. And you have people who die of old age when life comes to an end and the body just says at some point that it reckons it’s finished in this life and is going to quit. And then you notice a decline in the body and in the functioning of the body and then organs start to work a little less well; eventually the liver starts to work a less well and the kidneys start to work a little less well and you start building up waste products because they’re not working properly. You will get more and more tired, eventually you will sleep more, and eventually you will sleep well and in principle you won’t suffer much.” (Z4A03)

“But what we hope, of course, is that… well, this week is now very short. It’s only two days. But it’s ready now, in just a few days – we’ll have to see whether it’ll be in the weekend or a little earlier. But then that will happen one more time, the peak of the side effects is about a week and a half after the irradiation. So it will now take another week, maybe one and a half or two – it varies from person to person. Sure, but I expect those huge dips that you have had over the weekend are then over; I’m not expecting two more weekends. Because there’s no more new chemo and no new irradiation. So what I expect is that it can get even more tiring in the next week and a half and that your skin can get worse and the pain in the esophagus can get worse. But that huge dip in appetite will go differently than now in that weekend, I think.” (Z2R04)

**7. Giving information about emotional distress**

“That positions you more as someone with a disease, and that’s actually what you would rather not do.” (Z4A02)

“It’s already been discussed in the past, of course. On the one hand, that’s because of the severity of your lung problems, which mean you don’t actually do much. Not doing much then means you’re not going to feel much better, and at some point you get stuck in a vicious circle and one thing leads to another.” (Z3L02)

“In any case, she is quite optimistic and that’s important. Positive, even though some things might not be possible anymore, but you’re just… Because you could also just be gloomy, of course, and then things often don’t get better.” (Z3L05)

**Annex B. Affective communication quotes**

**1. Hope**

“Can we move forward in good spirits? There’s not much else we can do, right?” (Z1L03)

“It’s always nice to have something to look forward to.” (Z1L04)

“Yes, it is absolutely. It is going very well: the cancer is still small, so that can go well for a long time.” (Z3O04)

**2. Support**

“It isn’t all easy, I know? I’d like to be able to say otherwise, but that’s how it is and we must remain realistic, right?” (Z1L03)

“Because that’s the most important thing for us, and ultimately the most important thing for you.” (Z1L07)

“Sure. But that’s difficult, isn’t it?” (Z2R06)

**3. Reassurance**

“Fortunately, you have each other, which makes it more bearable.” (Z2L04)

“But every day that goes well is a day won, isn’t it?” (Z2R04)

**4. Empathy**

“But I understand that too, because that’s also what gives you the most joy.” (Z4A03)

“If you’re a patient yourself, you want to get the best information you can.” (Z3L05)

“No. But although it’s not new, it’s still annoying for you.” (Z4A04)

**5. Appreciation**

“That’s something you are already doing very well.” (Z2R05)

"I always think that’s nice to see with you: your face speaks volumes." (Z4A05)

“I’m convinced that you’ll be a great pianist!” (Z4A06)

**6. Emotional coping**

“Did you take that into account?” (Z1L03)

“Is there a bit of a fear that things could go wrong?” (Z3L02)
